# Spatial and spatio-temporal analysis for malaria hotspot identification: a scoping review protocol

**DOI:** 10.1136/bmjopen-2025-101375

**Published:** 2025-06-06

**Authors:** Gabriel Michel Monteiro, Roland Christel Sonounameto, Luc Salako Djogbenou, Luigi Sedda

**Affiliations:** 1 Faculté des sciences et Techniques, University of Abomey-Calavi, Cotonou, Benin; 2 African Institute for Mathematical Sciences Research and Innovation Centre, Kigali, Rwanda; 3 University of Abomey-Calavi Laboratory of Biomathematics and Forest Estimations, Cotonou, Benin; 4 University of Abomey-Calavi, Cotonou, Benin; 5 Department of Vector Biology, Liverpool School of Tropical Medicine, Liverpool, UK; 6 Lancaster Ecology and Epidemiology Group, Lancaster Medical School, Lancaster University, Lancaster, UK

**Keywords:** Malaria, Public health, Review, Geographical mapping

## Abstract

**Abstract:**

**Introduction:**

Malaria hotspots have been the focus of public health managers during the last two decades because of the potential elimination gains that can be obtained by targeting them. Advances in spatial technologies in the 20th century such as geographic information systems, remotely sensed satellite data on climate and ecology, and statistical methods for spatial cluster detection have enhanced our ability to map fine-scale patterns of malaria transmission. This led to the diversification of analytical approaches and a lack of consensus on methods and standardised indicators for malaria hotspot detection, raising challenges for comparing and synthesising findings across different studies. This review aims to fill this gap by identifying and summarising all publicly available peer-reviewed articles on spatial and spatio-temporal analytical approaches used to detect malaria hotspots while highlighting research gaps.

**Methods:**

This scoping review will follow the Joanna Briggs Institute Framework. A comprehensive search will be conducted in PubMed, Medline, Web of Science, Scopus and Embase using keywords related to malaria, hotspots and detection. Retrieved articles published between 1 January 2000 and 31 December 2024 in English or French will be uploaded to Covidence for screening. Empirical studies that apply spatial or spatio-temporal analytical methods to detect malaria hotspots will be included. Studies will be excluded if they rely solely on geographical visualisation without formal spatial analysis. Data extraction will be performed by two independent reviewers, with disagreements resolved by discussion. Data will be summarised using descriptive statistics and thematic analysis.

**Ethics and dissemination:**

This scoping review will involve the secondary analysis of published literature on malaria hotspot analysis; therefore, ethics approval is not required. The Preferred Reporting Items for Systematic Reviews and Meta-Analyses extension for scoping reviews checklist will be used to ensure transparency and methodological rigour in reporting. The findings will be disseminated through publication in a peer-reviewed journal and presented at scientific conferences via abstracts, oral or poster presentations.

**Trial egistration number:**

This review has been registered on the Open Science Framework under the DOI: https://doi.org/10.17605/OSF.IO/C8KUN.

**STRENGTHS AND LIMITATIONS OF THIS STUDY**

- This scoping review will apply a systematic search strategy across five major databases using predefined search terms and eligibility criteria.
- This protocol follows a structured and transparent methodology based on the Joanna Briggs Institute Framework, ensuring a rigorous synthesis of the literature on malaria hotspot analysis.
- Two reviewers will independently screen studies and extract data using Covidence to ensure consistency and reproducibility.
- As a scoping review, this study will not include a formal quality appraisal of the included studies, focusing instead on mapping available evidence to highlight trends, gaps and opportunities for future research.
- Limiting the literature search to English and French publications may exclude relevant studies published in other languages.

## Introduction

 Malaria, a life-threatening disease caused by *Plasmodium* parasites, continues to be a major public health concern, particularly in sub-Saharan Africa (SSA) where 90% of all malaria deaths occur.^1^ Heterogeneity in malaria transmission across space and time has long been recognised.^2^ The temporal variation in the disease’s transmission (also known as seasonality) is well documented in malaria-affected areas, where most malaria infections can occur in a few months.^3^ This is well exemplified in a study conducted in eastern Sudan, which demonstrated that approximately 90% of malaria cases in this region occur from September to November,^4^ while transmission remains very low or absent during months marked by drought,^5^ or by temperatures less suitable for the mosquitoes’ survival and malaria parasites’ development.^6^

Spatial heterogeneity in malaria transmission is also widely acknowledged, with studies showing variation at multiple geographic scales. At the household level, malaria risk may be influenced by proximity to vector breeding sites, housing structure or human behavioural factors, leading to clustering of infections within a few households.^7^ At the village level, malaria incidence can differ significantly between adjacent communities due to micro-environmental conditions, such as vegetation cover, water bodies or variations in vector control coverage within and between villages.^8^ Clustered malaria transmission patterns have also been demonstrated by ecological studies at regional^9^ and national levels.^10^

Together, these spatial and temporal variations in malaria transmission affect efforts for control and elimination, requiring optimal timing and geographic targeting of interventions.^11^ For this reason, the Global Technical Strategy for Malaria 2016–2030 adopted by the World Health Assembly in May 2015 recommends tailored and targeted interventions, thereby encouraging the identification of transmission hotspots to be targeted by geographically and population-specific interventions.^12^ This approach aims to ensure that interventions are efficient, context-specific and responsive to local epidemiological dynamics and emerging challenges.

By definition, malaria hotspots are specific geographical areas where malaria transmission rates are significantly higher than in surrounding regions.^13^ Malaria hotspots have been identified at various spatial scales, ranging from small household clusters and neighbourhoods to larger administrative units such as villages, districts or regions, depending on the spatial resolution of the study. In addition, it has been shown that a small proportion of the population living in hotspots bears the majority of parasite carriage in low transmission season, thereby fuelling onward transmission during high transmission period.^14^ This means that malaria hotspots may also demonstrate temporal stability or variability across different seasons, with some areas consistently experiencing higher transmission. Hence, identifying these high-burden areas within a locality is crucial, as it can facilitate the assessment of malaria transmission at a fine scale, and thus the deployment of geographically targeted interventions.^7^ Malaria hotspot identification can also help to prioritise limited resources available for malaria management in endemic settings.^15^

Earlier malaria risk mapping studies, especially from the mid-20th century, focused on large-scale risk assessment only without detailed spatial analysis^16^. Analyses of spatial heterogeneity in malaria transmission^17^ was aided by advances in spatial technologies, that is, geographic information systems (GIS), remotely sensed satellite data on climate and ecology,^18^ statistical methods for spatial cluster detection^19^ and exceedance probabilities^20^ . This led to the diversification of analytical approaches for hotspot analyses and a lack of consensus on the most effective methods and standardised indicators for their identification. This absence of methodological standardisation complicates the comparison of study findings, limits the ability to generalise results across different settings and hampers the formulation of evidence-based strategies for malaria control and elimination. A comprehensive synthesis is therefore needed to map current practices, identify methodological patterns, gaps, and guide the future development of consistent, evidence-based spatial methodologies for malaria hotspot detection.

Previous literature reviews on geospatial modelling of malaria risk have covered different aspects of the topic, including the identification of environmental risk factors of malaria transmission;^21^ the identification of factors that explain micro-epidemiological variation in risk;^22^ methods and covariates historically used for mapping malaria risk in SSA;^23^ the evolution of malaria risk mapping over the past decade and the improvements in data availability, computational power and methodological developments that have facilitated it.^24^ But to the best of our knowledge, there are no studies mapping the existing literature on malaria hotspot analysis or clustering despite the growing body of studies on the topic.

A scoping review is a common approach to evidence synthesis for researchers, public health professionals and policymakers.^25^ It is therefore a useful tool that can provide a comprehensive picture of the current state-of-the-art of research on malaria hotspot analysis. This scoping review aims to identify and present the available information regarding the methods and indicators used for malaria hotspot detection worldwide. This work has the following objectives: (1) identify, categorise and compare spatial and spatio-temporal methods used to detect malaria hotspots from 1 January 2000 to 31 December 2024; (2) assess the common indicators and covariates used, their spatial and temporal resolutions and hotspot definitions applied across studies and (3) identify methodological gaps and inconsistencies in the current literature and provide insightful recommendations for future research.

## Methods and analysis

This scoping review will adhere to the Joanna Briggs Institute framework for scoping reviews.^26^ It was initiated in January 2025 and is expected to be completed by December 2025.

### Inclusion criteria

This study will employ the Population, Concept and Context Framework to answer the research question^25^ as detailed below.

#### Population

This review will specifically focus on human malaria caused by *Plasmodium* parasites and does not include studies on malaria affecting non-human hosts. Therefore, the population of interest for this study is exclusively humans residing in regions where malaria infections occur.

#### Concept

The concept of interest for this scoping review is the spatio-temporal modelling techniques used to identify malaria hotspots, including but not limited to spatial clustering techniques (eg, SaTScan and Moran’s I), geostatistical models, exceedance probability mapping, generalised linear models, generalised additive models and machine learning approaches, such as random forests or boosted regression trees. In this review, we define key terms as follows:

- Malaria: is a disease caused by *Plasmodium* parasites, transmitted to humans through the bites of infected female *Anopheles* mosquitoes^1^;
- Heterogeneity: refers to diversity as opposed to homogeneity, which suggests uniform transmission or exposure to disease in the population.^27^ In malaria research, heterogeneity refers to the variation in the intensity of malaria transmission across space (spatial heterogeneity) and over time (temporal heterogeneity) in endemic areas. In this review, we will not impose a uniform or predefined threshold to define heterogeneity. Instead, we will extract and summarise how heterogeneity is defined, measured and operationalised in each individual study, including details about the spatial and/or temporal scale, data sources, statistical methods and threshold criteria used;
- Malaria foci: is “a defined and circumscribed locality situated in a currently or formerly malarious area and containing the continuous or intermittent epidemiological factors necessary for malaria transmission”^28^;
- Malaria hotspots: are areas located within malaria foci where malaria transmission intensity is above a threshold.^13^ Hotspots will be defined according to the geographical unit used in each study, such as household clusters or larger administrative units like villages, districts or regions.

#### Context

This review will focus solely on studies conducted in malaria countries focusing on the detection of malaria hotspots through spatio-temporal modelling, with implications for public health interventions. We will include only empirical studies that apply either spatial-only or spatio-temporal analytical techniques to detect malaria hotspots in real-world settings.

In light of the above, the review will include the following criteria:

1. Full-text journal articles.
2. Studies published in French or English between 1 January 2000 and 31 December 2024.
3. Studies with a clear focus on malaria hotspot identification.
4. Studies applying either spatial-only or spatio-temporal analysis techniques to investigate malaria risk in any geographical context not exceeding the national level.
5. Studies solely based on real-world malaria data as opposed to simulation or synthetic data.

The year 2000 has been chosen as the starting point to ensure consistency in the definition of malaria hotspots, a concept that emerged in the late 1990s. No exclusion will be made based on geography and modelling approaches. However, we will exclude studies that rely solely on GIS visualisation without any analytical insights—defined here as the application of spatial statistical methods, clustering algorithms or modelling frameworks to identify malaria hotspots. The review will also exclude conference proceedings, posters, protocols, book chapters, editorials, commentaries, brief reports and other non-peer-reviewed work, including grey literature. Additionally, multidisciplinary studies will be excluded if malaria hotspot identification is not among their primary objectives or if their detection and discussion of hotspots is limited to general mentions without any analytical insight. Studies published in languages other than English or French, and research unrelated to human malaria will also be excluded. The applied language restriction is based on the reviewers’ language capacity and the preliminary literature scoping, which showed that most relevant studies are published in English or French.

### Search strategy

We will undertake comprehensive literature searches in PubMed, Medline, Web of Science, Scopus and Embase databases. These five databases have been identified as an optimal set of databases expected to ensure adequate coverage of studies for this scoping review.^29^ The search strategy targeting peer-reviewed studies comprises three steps. First, a preliminary search of PubMed and Google Scholar was undertaken to retrieve a sample of the first 50 articles on the topic. The index terms used to describe these articles were assessed to identify the most relevant keywords for the subsequent search strategy. Google Scholar was used in an exploratory way during this phase to cross-check terminology, but it was not used in the formal literature search. Secondly, Medical Subject Headings (MeSH) terms were checked in the PubMed database. In the second step, searches using the keywords and index terms, such as malaria, *Plasmodium*, hotspot, cluster, outbreak and detection, will be performed. Boolean operators (AND, OR) will be used to combine these terms accordingly. In the third step, a thorough analysis of the reference lists of included studies will be undertaken to identify key articles that might have been missed in the previous steps. Although French-language articles will be considered for inclusion, preliminary scoping searches indicated that the addition of French-language keywords (eg, paludisme, foyer) did not significantly expand the retrieval of eligible studies. Therefore, the search strategy will primarily be based on English-language terms. The full search strategy for all five databases is presented in the online supplemental appendix 1 of the supplementary materials.

### Study selection

Results of the literature search will be uploaded (ie, citations, full titles and abstracts) to Covidence, which is a web-based application designed to streamline citation screening, study selection, data extraction and conflict resolution.^30^ It integrates with most reference management software, and it is endorsed by Cochrane, which further underscores its suitability for conducting this review. Two reviewers will screen the articles in a two-phase process: initial screening and full-text screening. For the initial screening process, the review team will read the abstracts of the studies and assess their relevance in light of the aforementioned inclusion criteria. To ensure consistency, both reviewers will first randomly select 10% of all the studies and independently review them. Any inconsistencies between the primary and secondary reviewers will be discussed to reach a consensus. Any strong disagreement that arises between the reviewers at either phase of the selection process will be resolved through discussion with all authors, and reasons for the exclusion of literature will be recorded and reported in the final scoping review. For the second phase of screening, full-text articles will be obtained for journal articles, and shortlisting will take place as in phase one. Explicit reasons for full-text exclusions (eg, wrong population, wrong study type, not a hotspot analysis) will be documented and reported. The results of the search and the screening process will be reported in the final scoping review and presented in a Preferred Reporting Items for Systematic Reviews and Meta-Analyses extension for scoping review (PRISMA-ScR) flow diagram.^31^

### Data extraction

Data extraction will be conducted using the Covidence platform. A structured data extraction form has been developed (see online supplemental appendix 2 in the supplementary materials) to systematically collect information from each eligible article. Extracted variables will include bibliographic details (such as authors, year, country, language and publication type), study aims and the malaria hotspot definition. In addition, specific attention will be given to input data sources, geographic and temporal scope and the analytical approach employed in each study, including the modelling framework (eg, spatial regression models, Bayesian models and machine learning approaches) and the type of hotspot detection method used (eg, SaTScan, Moran’s I and Getis-Ord Gi*); and the spatial and temporal resolution and the selection and treatment of predictor variables.

The extraction process will comprise a preliminary phase during which two reviewers will randomly select 10% of the papers and independently review them. Any disagreements that arise between the reviewers will be resolved through discussion with coauthors. The data extraction form will then be updated as needed, and extraction will be completed by reviewer GMM for all articles and checked for accuracy by another reviewer. Any modifications to the initial data extraction form will be reported in the final review.

### Data analysis and presentation

Extracted data will be analysed and presented as follows:

#### Descriptive analysis

Two reviewers will independently examine the extracted text and identify preliminary codes and emerging themes. They will then meet to develop a shared coding framework, which will be applied across all included articles by one author. Data recorded from articles will be analysed by GMM using R statistical software.^32^ We will conduct a descriptive summary covering the number of studies, their design, year of publication, covariates, administrative level of analysis, population characteristics and reported public health implications.

#### Spatial visualization

A trend plot showing the number of studies based on their year of publication will be produced, and countries of publication will be presented on a choropleth map depicting the distribution of studies. This map will allow the identification of areas with knowledge gaps. Additional figures will focus on the methodological traits (including but not limited to types of data sources and spatial and temporal resolutions) of included studies and will serve to highlight the evolution of modelling approaches over time.

#### Thematic synthesis

We will conduct a thematic analysis to organise the characteristics of the studies into categories related to hotspot detection. This will include types of outcome measures, data sources, modelling frameworks (eg, spatial-only or spatio-temporal), hotspot definitions and the predictors used. These findings will be presented using summary tables and figures. Finally, a discussion of the practical implications for malaria research, modelling and public health decision-making will be carried out while highlighting gaps and future research needs related to malaria hotspot analysis.

As this is a scoping review, a formal quality appraisal of included studies will not be conducted. This methodological limitation, along with the absence of critical review of the methodologies of included articles and the restriction to English- and French-language publications, may affect the quality of the synthesis and lead to the exclusion of potentially relevant studies. These limitations will be transparently reported and discussed in the final review.

## Ethics and dissemination

This review is based on the secondary analysis of published literature, and therefore ethics approval is not required. The report of the scoping review will follow the PRISMA-ScR checklist to ensure a structured and transparent synthesis of findings. The results will be shared through publication in a peer-reviewed journal and presented at conferences through abstracts and oral or poster presentations.

### Patient and public involvement

Patients or the public were not involved in the design, conduct, reporting or dissemination plans of our research.

## Supplementary material

10.1136/bmjopen-2025-101375 online supplemental file 1
