# Supplementary material for: Spatial and spatio-temporal analysis for malaria hotspot identification: a scoping review protocol
Source: BMJ Open. 2025 Jun 6;15(6):e101375. doi: 10.1136/bmjopen-2025-101375 (PMC12161422; doi:10.1136/bmjopen-2025-101375)
Supplement: online supplemental file 1 [file bmjopen-15-6-s001.docx]

# Appendices

## Appendix I: Search strategy

| Database | Date of search | Date limits | Search terms | Number of publications retrieved |
| --- | --- | --- | --- | --- |
| PubMed and MEDLINE | 04/12/2024 | 01/01/2000 to 04/12/2024 | ((malaria[Title/Abstract] OR plasmodium[Title/Abstract]) AND (hotspot[Title/Abstract] OR cluster*[Title/Abstract] OR outbreak[Title/Abstract])) AND (model*[Title/Abstract] OR identification[Title/Abstract] OR detection[Title/Abstract] OR analysis[Title/Abstract]) | 2237 |
| Embase | 04/12/2024 |  | (malaria hotspot.ti,ab.) OR (plasmodium hotspot.ti,ab.) | 35 |
| Scopus | 04/12/2024 |  | TITLE-ABS-KEY ( ( malaria OR plasmodium ) AND ( hotspot ) AND ( mapping OR modelling OR analysis OR clustering OR detection OR identification ) ) AND PUBYEAR > 2002 AND PUBYEAR < 2025 | 380 |
| Web of science | 04/12/2024 |  | (TI=((( malaria OR plasmodium ) AND ( hotspot ) AND ( mapping OR modelling OR analysis OR clustering OR detection OR identification ) ) )) OR AB=((( malaria OR plasmodium ) AND ( hotspot ) AND ( mapping OR modelling OR analysis OR clustering OR detection OR identification ) ) ) | 311 |

## Appendix II: Data extraction form

| Section | Data |
| --- | --- |
| Bibliographic information | - Study ID - Authors, year - Country - Full title - Language - DOI - Type of publication (journal article, book chapter, grey literature) |
| Definition | - Definition of malaria hotspots |
| Aims | - Hotspot/clusters detection focus and purpose (e.g. intervention planning, risk mapping) |
| Methodology | - Study period (start date – end date) - Geographical scope of the study (City/cities the study was conducted) - Study population/spatial unit (e.g. household, national, province, district facility, malaria case, census tract, other) - Geographic scope of hotspot (e.g., national, regional, village-level, 1 km) - Temporal scope of hotspot (e.g., monthly seasonal, perennial, or specific years) - Data sources used: multiples sources employed, different study designs and sampling frames employed - Outcomes variables - Clinical outcomes (e.g., malaria incidence, prevalence). - Entomological metrics (e.g., vector density, sporozoite rates). - Threshold for definition of hotspot (e.g., based on malaria incidence thresholds, vector abundance, predicted risk, or other criteria) - Predictors(s) selection approach - Modeling Framework - Specific spatial or spatio-temporal analysis techniques: Bayesian models, machine learning, SaTScan, etc. - hotspot detection approach (e.g. Moran I or Getis-Ord Gi*, etc) |
| Conclusions and recommendations | - Policy implications - Unexpected results - Modelling gap(s) - Challenges in hotspot identification |
