# Supplementary material for: Spatial and spatio-temporal analysis for malaria hotspot identification: a scoping review protocol
Source: BMJ Open. 2025 Jun 6;15(6):e101375. doi: 10.1136/bmjopen-2025-101375 (PMC12161422; doi:10.1136/bmjopen-2025-101375)
Supplement: Supplementary file 2 [file bmjopen-2025-101375.reviewer_comments.pdf]

PEER REVIEW HISTORY

BMJ Open publishes all reviews undertaken for accepted manuscripts. Reviewers are asked to complete a checklist review form and are provided with free text boxes to elaborate on their assessment. These free text comments are reproduced below.

ARTICLE DETAILS

Title (Provisional)

Spatial and spatio-temporal analysis for malaria hotspots identification: a scoping review protocol

Authors

Monteiro, Gabriel Michel; Sonounameto, Roland Christel; Djogbenou, Luc Salako; Sedda , Luigi

---

VERSION 1 - REVIEW

**Reviewer**	**1**
**Name**	**McGee, Kathleen**
**Affiliation**	**London School of Hygiene & Tropical Medicine**
**Date**	**04-Apr-2025**
**COI**	**None**

This is a very interesting and timely review that has the potential to make a great contribution to the literature and will be a very useful (and interesting!) resource for researchers and policymakers. However, to reach its full potential, and before the manuscript can be considered for publication, a number of important revisions are needed. These include improvements to language and grammar, greater clarity and specificity in the methods, and a more robust articulation of the review's rationale and key concepts. These are important refinements that I think will significantly strengthen the manuscript.

- General comments:
    - This is a very interesting and timely review that has the potential to make a great contribution to the literature and will be a very useful (and interesting!) resource for researchers and policymakers.
    - Before the manuscript can be considered for publication, a number of important revisions are needed.
        - The manuscript—particularly the abstract and introduction—requires careful revision for grammar, sentence structure, and overall clarity. While the scientific content is promising, language issues significantly hinder readability.
        - Specificity. Key aspects of your methods and definitions need to be made more explicit. This includes further expanding on your very broad definitions of hotspot and heterogeneity, being more explicit on what

studies you will or won’t include, and ensuring consistency between what is stated in the abstract and what is described in the main text.

- List of authors
    - Rwanda misspelled
- Abstract
    - Major comments:
        - Line 20: “while pinpointing research gaps and methodological issues”- research gaps ok- but methodological issues? Nowhere in the manuscript do you discuss how you would critically appraise the methodologies and identify issues. This is not addressed in your aims and objectives, nor is it discussed in your analytical plan. Either review that statement in your abstract or you need to clarify in the remainder of your manuscript how methodological issues will be identified and reported.
        - Line 22:” using keywords related to malaria hotspots”. Your search strategy as defined later in your manuscript and in the appendix actually relates to malaria, hotspots, AND detection. You’re missing the 3rd part to your search logic.
        - Line 28–29: “Findings will be disseminated through peer-reviewed journals...” — make it singular “a peer-reviewed journal” unless you plan on publishing in multiple journals which you need to justify and explain then in your main manuscript.
    - Minor comments:
        - Line 13: “Malaria hotspots has been” - should be “have been”
        - Line 13: Remove “to” at the end
        - Line 14: “Advances of spatial technologies” - “Advances in spatial technologies.”
        - Line 15: “i.e.” should be “e.g.” if you’re giving examples.
        - Line 15: Remove “(GIS)” if the acronym isn’t used again later in abstract
        - Line 16: “Enhanced the ability” - rephrase: “have enhanced our ability to map...”
        - Line 21: Remove JBI and the parentheses- just keep Joanna Briggs Institute.
        - Line 23: “Retrieve articles...” - “Retrieved articles...”
        - Line 24: “Data extraction to be performed...” - better as: “will be performed by two independent reviewers...”
        - Lines 21-24: You’ve only addressed the search, and data extraction. You should also briefly explain how you plan to analyze or synthesize the evidence.
        - Line 29: Add period at the end.
- Strengths and Limitations:
    - Major comments:
        - I think you’re underselling the contribution. In addition to synthesizing evidence, you are also identifying research gaps and providing recommendations (as stipulated by your aims—both of these are additional contributions that your statement doesn’t capture.

        - Ok no formal quality appraisal. This is not mentioned in your main manuscript. You need to address limitations not just in this segment but in the main manuscript itself.
        - Are you also doing a critical review of the methodologies themselves as mentioned in your abstract? If yes, that needs to be clearer. If not, that is a limitation that needs to be explicitly said.
    - Minor comments:
        - Line 33: “One of the first scoping review” - should be “scoping reviews.”
        - Line 36: “Ensuring a rigorous...” - redundant with “follows a rigorous... method.” Trim.
        - Line 36: again no need for the acronym here- keep those for the main manuscript.
- Introduction:
    - Major comments:
        - Lines 60-65: you should be clearer on the geographical unit or scale a malaria hotspot can represent. ‘You mention “high-burden areas within a locality,” but it’s not specified whether these areas refer to regions, districts, towns, neighbourhoods, or household clusters. Since the spatial resolution can significantly vary and influence how hotspots are identified it would strengthen the text to make this variation explicit.
        - Lines 60-65: Is there also a temporal aspect to the definition of hotspots? Your paragraph focuses almost entirely on geography.
        - Line 72: This sentence introduces what appears to be the central rationale for your review — that the diversity in methodologies has led to a lack of consensus on effective approaches and standardized indicators for malaria hotspot detection. That sentence is doing a lot of heavy lifting but, it’s currently too brief, unsupported (no references?) and not sufficiently unpacked. Expand this sentence- why does that matter? You need to ground the purpose of your review and clearly justify its relevance.
    - Minor comments:
        - Line 46: Sentence “Malaria transmission shows a heterogeneous has long been...” is grammatically incorrect. Suggest: “Heterogeneity in malaria transmission across space and time has long been recognized.”
        - Line 48: “Malaria episodes” — clarify what you mean. ‘Episode’ is not sufficiently specific. Do you mean infections?
        - Line 50–51: Rephrase for clarity.
        - Line 52: “Spatial heterogeneity in malaria heterogeneity...” — remove 2nd heterogeneity.
        - Line 53: make sure your commas are consistently put either before or after citations.
        - Line 54: “Spatiotemporal heterogeneous distribution affect...”- decide if you’re spelling spatiotemporal with or without a ‘-‘ and stay consistent across the manuscript
        - Across: please reduce your use of thereby and therefore.
        - Lines 60-62: The link between your first sentence defining malaria hotspots- and your second sentence- indicating the role of small

proportions of populations in fueling later transmissions is not clear. These 2 thoughts are disconnected and need a more explicit link.

- Line 63: "Facilitates" should be "facilitate."
- Line 66–67: "Most malaria risk mapping studies..." - awkward phrasing. Suggest: "Earlier malaria risk mapping studies, especially from the mid-20th century, focused on large-scale patterns and lacked fine spatial resolution.
- Line 68: "Publication more fine-space studies..." - do you mean fine-scale?
- Line 69: "Advances of spatial technologies" - again, should be "advances in."
- Line 70: "And exceedance probabilities" — reference?
- Line 71: "This leads to..."- should be in the past tense
- Line 73: "Reviews on geospatial modelling... has covered". Should be: "...have covered."
- Selection of factors- selection is not the appropriate word.
- Line 80: "It is therefore a powerful tool..." — powerful is a bit exaggerated. Useful tool.
- Line 81: "State-of-art" should be "state-of-the-art."
- Line 85: "Identify research gap" - should be "gaps."
- Line 85: "Future researches" - should be "future research."

Please note- I will not provide further detailed corrections to grammar or sentence structure through the rest of the manuscript. The introduction, particularly, contains too many issues that hinder readability and clarity.

- Methods and analysis
  - Major comments:
    - Concept
      - Line 97. Not etc. This is a critical part of your review- so these should be listed and defined to your best ability at this stage. Instead of etc, you should write "including but not limited to: and then list all the approaches, not just a few of them.
      - It's unclear whether studies that only apply spatial methods (i.e., analyze geographic variation at a single point in time) will be included, or if only studies with both spatial and temporal dimensions qualify. You hint to it in the last sentence of this section and in your title – but it needs to be explicitly said.
      - You might want to specify if you are only looking at empirical studies or if you will include modelling studies using simulation data.
      - Heterogeneity. That last sentence should specify that it refers to variation in intensity of malaria transmission in both a geographical sense and a temporal sense.
      - Heterogeneity. Also, this definition is pretty broad. Heterogeneity may be measured differently across studies -depending on scale, data sources, measurement and threshold criteria. I can see in your data extraction table that you've considered these aspects but you haven't made that explicit in your manuscript. So be explicit and clarify whether you will apply a uniform and

        quantifiable threshold to define heterogeneity or whether you will extract and summarize the criteria used by individual studies (likely the latter- but you need to state it).
        - Hotspots: As raised earlier, clarify the spatial unit(s) being considered. Since this is a scoping review, you don't need to restrict it — but you should state that hotspot will be defined based on the geographical unit used in each study (e.g., village, district, administrative level).
    - Minor Comments:
        - You don't use the JBI acronym in the remainder of the manuscript so there is no need for it.
- Search strategy:
    - Major comments:
    - Minor comments:
        - Since you're also considering French articles I wonder if you shouldn't also consider adding a few French-word equivalents to your search strategy or run a separate search strategy entirely in French. For example adding paludisme to malaria OR plasmodium, or foyer to hotspot OR cluster OR outbreak. I'm not sure this is necessary – just food for thought.
- Study selection:
    - Major comments:
    - Minor comments:
        - I would add that, conforming to guidelines, you will provide explicit reasons for full-text exclusions (e.g., wrong population, wrong study type, not a hotspot analysis, not spatially disaggregated, etc.)
- Data extraction:
    - Major comments:
        - I find this paragraph to be pretty slim – and it's a really important one. There needs to be more explicit description of what you will extract- especially under 'analytical approach'. I would add a few sentences there just expanding on that category.
    - Minor comments:
- Data analysis
    - Major comments:
        - There should definitely be a sentence or 2 here on limitations.
    - Minor comments:
        - Very interesting analysis. The paragraph currently gives the impression that trend plots and maps will be used for only number of studies and countries of publication. Given your interest in methods, data sources, and spatial resolution, you might consider revising this paragraph to reflect a broader use of visual tools to show the evolution of modelling approaches and spatial resolutions over time.
- Ethics:
    - Results will be shared through publication in peer-reviewed journals- why the plural? are you planning on writing more than 1 article out of this scoping review? If so that needs to be said somewhere with a clear description of what each article would address. If not then change that to the singular.

**Reviewer**	**2**
**Name**	**Mahmud , Mohd Amierul Fikri**
**Affiliation**	**Institute for Public Health, Malaysia, Center for Communicable Disease Research**
**Date**	**14-Apr-2025**
**COI**	**None**

I think this is a relevant and timely topic focusing on spatial and spatio-temporal analytical approaches in malaria hotspot detection. I`ve summarized a few suggestions for improvements:

1. Abstract
   - Kindly revise the sentences to improve grammar and ensure clearer expression. Eg;"...because of to the potential..” incorrect; should be "because of the potential..." Please check others.
   - Add briefly the inclusion/exclusion criteria

2. Introduction
   - Structure and Logical Coherence
     1) Temporal vs. Spatial Heterogeneity:
     The section starts with a mix of temporal and spatial variation without clearly separating the two concepts.
     Suggest: Dedicate one paragraph to temporal heterogeneity and another to spatial, then combine to explain spatiotemporal heterogeneity.

     2) Flow of Evidence & Citations:
     While many studies are cited, the relevance of some is not always clearly tied to the narrative.
     Suggest briefly interpreting what each cited study contributes (e.g., “as shown in Sudan, seasonal variation impacts intervention timing”).

   - Rationale of the scoping review
     1) The rationale is somewhat buried in a long paragraph. This dilutes the impact.
     2) You mention that methods have diversified, but not why that matters enough for a dedicated review?
     3) Lines 81–85 say the review will *“identify and present available information”*—this could be more specific. Eg: This review will categorize and compare spatial and spatio-temporal methods, assess common indicators, and highlight trends or inconsistencies in hotspot definitions and applications…..

3. Methods
   - The PCC framework explanation (Population, Concept, Context) is informative, but the subsections would benefit by bullet formatting or clearer subheadings (e.g., bold or italicized "Population", "Concept", "Context") to improve readability.
   - No Boolean operators or filters mentioned

- Reference to Google Scholar without justification as Google Scholar lacks transparency and indexing control.
- No mention of publication management software. Add that the results will be imported into Covidence or similar software.
- Exclusion of studies using only GIS visualization- Good decision. Clarify what constitutes "analytical insight" (e.g., statistical modelling vs descriptive mapping)?
- Language limitation to English and French- Reasonable, but mention the rationale; likely due to author capacity or resource constraints.
- “Multidisciplinary studies with only a minor or superficial focus…” - Consider specifying what defines a “superficial focus”; is it word count? lack of quantitative data?

4. Data extraction
   - Lack of information on which variables will be extracted
     Readers and reviewers need to know what type of information the authors plan to synthesize (e.g., study design, modelling techniques, indicators, outcomes).

5. Data analysis and presentation
   - The analysis plan feels somewhat scattered and blends narrative, statistical, and visual approaches in one paragraph.
     Suggestion: Break into clearly labelled subsections for..
     Descriptive Analysis, Spatial Visualization, Thematic Synthesis and Implication Mapping

   - Consider adding a PRISMA Flow Diagram (if not already planned)

   - Please ensure that the data analysis and presentation address the objectives of the scoping review, which include: 1) Identifying and Describing Analytical Methods, 2) Evaluating the Covariates Employed, and 3) Identifying Research Gaps and Recommendations for Future Research.

VERSION 1 - AUTHOR RESPONSE

Reviewer 1

Regarding the comments from Reviewer 1, we have addressed all of them as shown in the new submitted article (please see main document). In particular (focusing on the major comments):

- General comments
  - *The manuscript—particularly the abstract and introduction—requires careful revision for grammar, sentence structure, and overall clarity. While the scientific content is promising, language issues significantly hinder readability*

  Response: We thank the reviewer for all important observations about grammar and clarity. The abstract and introduction have been carefully revised for grammar, sentence structure, and overall readability to enhance clarity.

- *Specificity. Key aspects of your methods and definitions need to be made more explicit. This includes further expanding on your very broad definitions of hotspot and heterogeneity, being more explicit on what studies you will or won't include, and ensuring consistency between what is stated in the abstract and what is described in the main text.*

  Response: We have clarified definitions of "malaria hotspots" and "heterogeneity," and made explicit what studies will be included or excluded. Inconsistencies between the abstract and main text have been resolved. [lines 121-127 and 130-132]

- *Rwanda misspelled*

  Response: Thank you. Correct spelling of "Rwanda" in the author affiliation section has been updated.
- Abstract
  - *Line 20: "while pinpointing research gaps and methodological issues"- research gaps ok- but methodological issues? Nowhere in the manuscript do you discuss how you would critically appraise the methodologies and identify issues. This is not addressed in your aims and objectives, nor is it discussed in your analytical plan. Either review that statement in your abstract or you need to clarify in the remainder of your manuscript how methodological issues will be identified and reported.*

    Response: We removed "methodological issues" from the abstract to avoid suggesting formal critique not present in the methodology. [line 21]

  - *Line 22:" using keywords related to malaria hotspots". Your search strategy as defined later in your manuscript and in the appendix actually relates to malaria, hotspots, AND detection. You're missing the 3rd part to your search logic.*

    Response: We revised this sentence to reflect that our search terms relate to malaria, hotspots, and detection. [lines 23-24]

  - *Line 28–29: "Findings will be disseminated through peer-reviewed journals…" — make it singular "a peer-reviewed journal" unless you plan on publishing in multiple journals which you need to justify and explain then in your main manuscript.*

    Response: We have revised this phrase to mean a single peer-reviewed journal. [line 32]

  - Additional grammatical issues mentioned in minor comments

    Response: All grammatical and structural suggestions have been implemented as follows:

    - "Malaria hotspots has been" corrected to "Malaria hotspots have been" [line 14]
    - "to" removed [line 14]
    - "Advances of spatial technologies" corrected to "Advances in spatial technologies" [line 15]
    - "i.e." corrected to "such as" [line 16]
    - the acronym "(GIS)" has been removed [line 16]
    - "Enhanced the ability" rephrased to "have enhanced our ability…" [line 17]
    - The acronym JBI and the parentheses have been removed [line 22]
    - "Retrieve articles…" corrected to "Retrieved articles…" [line 24]
    - "Data extraction to be performed…" corrected to "will be performed by two independent reviewers…" [line 27]
    - A period has been added at the end of the sentence. [line 33]

- *You've only addressed the search, and data extraction. You should also briefly explain how you plan to analyze or synthesize the evidence.*

Response: The abstract has been updated to mention that extracted evidence will be summarized using descriptive statistics and thematic analysis. [line 28]

- Strengths and limitations of this study
  - Major comments
    - *I think you're underselling the contribution. In addition to synthesizing evidence, you are also identifying research gaps and providing recommendations (as stipulated by your aims—both of these are additional contributions that your statement doesn't capture.*

Response: We have actually focused the points of this section to align with journal policy by removing novelty and ensuring all points are method-focused.

    - *Ok no formal quality appraisal. This is not mentioned in your main manuscript. You need to address limitations not just in this segment but in the main manuscript itself.*

Response: Absence of formal quality appraisal and scope limitations are reflected in both this section and the Methods. [lines 43 and 219]

    - *Are you also doing a critical review of the methodologies themselves as mentioned in your abstract? If yes, that needs to be clearer. If not, that is a limitation that needs to be explicitly said.*

Response: We now clearly stated the limitations of the review at the end of the Methods section, including the absence of critical review of the methodologies of included articles. [lines 219-222]

  - Grammatical issues mentioned in minor comments

Response: All grammatical issues highlighted has been addressed as follows:

    - The first point of the 'Strengths and limitations of this study' section has been revised and the sentence now reads: "This scoping review will apply a systematic search strategy across five major databases using predefined search terms and eligibility criteria". [lines 37-38]
    - The redundant use of the word 'rigorous' has now been trimmed. [lines 39-40]
    - The acronym 'JBI' has been removed. [lines 39]
- Introduction
  - Major comments:
    - *Lines 60–65: you should be clearer on the geographical unit or scale a malaria hotspot can represent. 'You mention "high-burden areas within a locality," but it's not specified whether these areas refer to regions, districts, towns, neighbourhoods, or household clusters. Since the spatial resolution can significantly vary and influence how hotspots are identified it would strengthen the text to make this variation explicit.*

Response: We have expanded the definition of malaria hotspots to indicate that they can range from small household clusters and neighborhoods to larger administrative units such as villages, districts, or regions, depending on the spatial resolution of each study. [lines 68-71]

- *Lines 60–65: Is there also a temporal aspect to the definition of hotspots? Your paragraph focuses almost entirely on geography.*

Response: We explicitly demonstrated that malaria hotspots may also demonstrate temporal stability or variability across seasons, providing a more complete definition. The manuscript in its current version dedicates one paragraph to temporal heterogeneity and another to spatial, then combine to explain spatiotemporal heterogeneity. [lines 48-63]

- *Line 72: This sentence introduces what appears to be the central rationale for your review — that the diversity in methodologies has led to a lack of consensus on effective approaches and standardized indicators for malaria hotspot detection. That sentence is doing a lot of heavy lifting but, it's currently too brief, unsupported (no references?) and not sufficiently unpacked. Expand this sentence- why does that matter? You need to ground the purpose of your review and clearly justify its relevance.*

Response: We expanded the rationale paragraph to explain that the lack of methodological standardization complicates comparison, limits generalizability, and hinders evidence-based intervention design. [lines 83-88]

- Minor comments:
  - *Line 46: Sentence "Malaria transmission shows a heterogeneous has long been…" is grammatically incorrect. Suggest: "Heterogeneity in malaria transmission across space and time has long been recognized."*

Response: We revised the sentence to: "Heterogeneity in malaria transmission across space and time has long been recognized." [lines 49-50]

  - *Line 48: "Malaria episodes" — clarify what you mean. 'Episode' is not sufficiently specific. Do you mean infections?*

Response: We clarified the wording by replacing "episodes" with "infections." The revised sentence now refers to "malaria infections," ensuring precision and eliminating potential ambiguity. [line 51]

  - *Line 50–51: Rephrase for clarity.*

Response: We rephrased this part for better clarity. It now reads: "This is well exemplified in a study conducted in eastern Sudan, which demonstrated that approximately 90% of malaria cases in this region occurs from September to November, while transmission remains very low or absent during months marked by drought, or by temperatures less suitable for the mosquitoes' survival and malaria parasites development." [lines 51-54]

  - *Line 52: "Spatial heterogeneity in malaria heterogeneity…" — remove the second 'heterogeneity'.*

Response: We removed the redundant word. The corrected phrase now reads: "Spatial heterogeneity in malaria transmission is also widely acknowledged," improving readability and avoiding repetition. [line 56]

  - *Line 53: Make sure your commas are consistently placed before or after citations.*

Response: We carefully reviewed the entire Introduction section to standardize comma placement. Commas are now consistently placed after citation parentheses.

- *Line 54: "Spatiotemporal heterogeneous distribution affect…"- decide if you're spelling spatiotemporal with or without a '-' and stay consistent across the manuscript*

Response: We chose the spelling "spatio-temporal" and applied it consistently throughout the manuscript, including the Introduction section.

- *Across the Introduction: please reduce your use of "thereby" and "therefore."*

Response: We revised the sentences throughout the Introduction to reduce the overuse of "thereby" and "therefore." In cases where it was necessary to retain logical connectors, we alternated with other linking words such as "thus" or "hence". [lines 74 and 75]

- *Lines 60–62: The link between your first sentence defining malaria hotspots and your second sentence about small proportions of populations fueling later transmissions is not clear.*

Response: We improved the logical connection between the two ideas. The paragraph now explicitly explains that individuals residing in hotspots maintain parasite reservoirs during low-transmission seasons, which fuels onward transmission during high-transmission periods. [lines 68-73]

- *Line 63: "Facilitates" should be "facilitate."*

Response: We corrected the verb agreement. The sentence now reads: "...as it can facilitate the assessment of malaria transmission at fine-scale..." [line 75]

- *Line 66–67: "Most malaria risk mapping studies…" - awkward phrasing. Suggest: "Earlier malaria risk mapping studies, especially from the mid20th century, focused on large-scale patterns and lacked fine spatial resolution.*

Response: We rephrased the sentence to improve readability. It now reads: "…earlier malaria risk mapping studies, especially from the mid-20th century, focused on large-scale risk assessment without detailed spatial analysis…" [line 78-79]

- *Line 68: "Publication more fine-space studies..." — do you mean fine-scale?*

Response: We corrected the phrase to "fine-scale studies," which is the appropriate terminology. [line 80]

- *Line 69: "Advances of spatial technologies" — again, should be "advances in."*

Response: "Advances of spatial technologies" corrected to "Advances in spatial technologies". [lines 80-81]

- *Line 70: "And exceedance probabilities" — reference?*

Response: We added a reference to Mategula et al. (2024) to support the mention of exceedance probabilities, which are discussed within model-based geostatistics for disease mapping. [line 82]

- *Line 71: "This leads to..." — should be past tense.*

Response: We changed "This leads to" to "This led to" to maintain correct tense alignment with the historical development described. [line 83]

- *Line 73: "Reviews on geospatial modelling… has covered" should be "have covered."*

Response: We corrected the verb agreement. The sentence now reads: "Previous literature reviews on geospatial modelling of malaria risk have covered..." [line 89]

- *Selection of factors — "selection" is not the appropriate word.*

Response: We replaced "selection of factors" with "identification of factors" to better reflect the process described in the cited reviews. [line 89]

- *Line 80: "It is therefore a powerful tool..." — powerful is a bit exaggerated.*

Response: We moderated the language and revised the sentence to: "It is therefore a useful tool that can provide a comprehensive picture..." [line 97]

- *Line 81: "State-of-art" should be "state-of-the-art."*

Response: We corrected the phrasing to "state-of-the-art" throughout the manuscript. [line 97]

- *Line 85: "Identify research gap" — should be "gaps."*

Response: We corrected the phrase to "identify methodological gaps" reflecting the likelihood of multiple gaps. [line 102]

- *Line 85: "Future researches" — should be "future research."*

Response: We corrected the term to "future research," which is now grammatically correct. [line 103]

- Methods and analysis
  - Mayor comments:
    - *Line 97: Not etc. This is a critical part of your review- so these should be listed and defined to your best ability at this stage. Instead of etc, you should write "including but not limited to: and then list all the approaches, not just a few of them.*

Response: We agree that the suggested precision is necessary here. In the revised manuscript, we replaced "etc." with a more detailed enumeration of the analytical approaches to be considered, including spatial clustering methods (e.g., SaTScan, Moran's I), geostatistical modeling, exceedance probability methods, and machine learning-based classification models. [lines 115-118]

- *It's unclear whether studies that only apply spatial methods (i.e., analyze geographic variation at a single point in time) will be included, or if only studies with both spatial and temporal dimensions qualify. You hint to it in the last sentence of this.*

Response: We appreciate this important clarification request. We have now explicitly stated that both spatial-only and spatio-temporal studies will be eligible for inclusion. This aligns with the broad scope of the review aiming to capture the diversity of methods used to detect malaria hotspots. [line 136]

- *You might want to specify if you are only looking at empirical studies or if you will include modelling studies using simulation data.*

Response: Thank you for this observation. We have clarified that studies using only real-world malaria data to detect malaria hotspots will be included. [lines 136-137 and 144]

- *Heterogeneity. That last sentence should specify that it refers to variation in intensity of malaria transmission in both a geographical sense and a temporal sense.*

Response: We fully agree. In the revised manuscript, we made explicit that heterogeneity will refer to both spatial (geographic) and temporal variation in malaria transmission intensity. [line 123]

- *Heterogeneity. Also, this definition is pretty broad. Heterogeneity may be measured differently across studies -depending on scale, data sources, measurement and threshold criteria. I can see in your data extraction table that you've considered these aspects but you haven't made that explicit in your manuscript. So be explicit and clarify whether you will apply a uniform and quantifiable threshold to define heterogeneity or whether you will extract and summarize the criteria used by individual studies (likely the latter- but you need to state it).*

Response: We thank the reviewer for highlighting this. We have clarified that we will extract and summarize the definitions, thresholds, and measurement criteria used in individual studies, rather than imposing a uniform operational definition of heterogeneity. [lines 124-127]

- *Hotspots: As raised earlier, clarify the spatial unit(s) being considered. Since this is a scoping review, you don't need to restrict it — but you should state that hotspot will be defined based on the geographical unit used in each study (e.g., village, district, administrative level).*

Response: This has been addressed. We explicitly state that hotspots will be interpreted based on the spatial unit defined within each study (e.g., household cluster, village, district, administrative region). [lines 131-132]

- Minor comments:
  - *You don't use the JBI acronym in the remainder of the manuscript so there is no need for it.*

Response: We have removed the acronym (JBI) after the first occurrence of "Joanna Briggs Institute" in the section.

- Search strategy
  - *Since you're also considering French articles I wonder if you shouldn't also consider adding a few French-word equivalents to your search strategy or run a separate search strategy entirely in French. For example adding paludisme to malaria OR plasmodium, or foyer to hotspot OR cluster OR outbreak. I'm not sure this is necessary – just food for thought.*

Response: We thank the reviewer for this thoughtful suggestion. However, we decided not to add French-language keywords such as "paludisme" or "foyer" to our search strategy. During preliminary scoping searches, we observed that studies focusing on malaria hotspot identification published in French were extremely limited. Most relevant studies in malaria-endemic regions, including French-speaking countries, were published in English, and the addition of French terms did not meaningfully expand the pool of eligible articles. Therefore, we maintained an English-words-based search strategy while including French-language studies when identified through English keywords. We have explicitly mentioned this in the current version of the manuscript. [lines 167-170]

- Study selection
  - *I would add that, conforming to guidelines, you will provide explicit reasons for full-text exclusions (e.g., wrong population, wrong study type, not a hotspot analysis, not spatially disaggregated, etc.).*

Response: We have incorporated this important clarification. In the revised manuscript, we state that reasons for full-text exclusions (e.g., wrong population, wrong study type, not hotspot analysis) will be recorded and reported. [lines 182-183]

- Data extraction

Mayor comments:

  - *I find this paragraph to be pretty slim – and it's a really important one. There needs to be more explicit description of what you will extract especially under 'analytical approach'. I would add a few sentences there just expanding on that category.*

  Response: We thank the reviewer for emphasizing the importance of clearly specifying our data extraction strategy. In the revised manuscript, we have expanded the Data Extraction section to explicitly describe the full range of variables that will be extracted. In particular, we detailed the information to be collected under "analytical approach," including the type of spatial or spatiotemporal modeling frameworks used, the hotspot detection methods applied (e.g., cluster detection, spatial autocorrelation statistics), data sources, definitions and thresholds for hotspot delineation, and types of outcome and predictor variables analyzed. [lines 188-193]

- Data analysis
  - Major comments:
    - *There should definitely be a sentence or 2 here on limitations.*

  Response: We have addressed this by adding a sentence explaining that, as a scoping review, the study will not perform a formal quality appraisal, and this limitation will be acknowledged in the discussion. We also pinpointed limitations due to the restriction of publication language of studies to be included to English and French. [lines 219-222]

  - Minor comments:
    - *Very interesting analysis. The paragraph currently gives the impression that trend plots and maps will be used for only number of studies and countries of publication. Given your interest in methods, data sources, and spatial resolution, you might consider revising this paragraph to reflect a broader use of visual tools to show the evolution of modelling approaches and spatial resolutions over time.*

  Response: We have revised all the Data analysis section to reflect that a diversity of visualizations will also show trends in analytical methods, spatial resolutions, and types of covariates over time.

- Ethics and dissemination
  - *Results will be shared through publication in peer-reviewed journals- why the plural? are you planning on writing more than 1 article out of this scoping review? If so that needs to be said somewhere with a clear description of what each article would address. If not then change that to the singular.*

  Response: Thank you for mentioning that. We have revised the statement to reflect that findings will be published in a single peer-reviewed journal. [line 226]

Reviewer 2

1. Abstract

- Kindly revise the sentences to improve grammar and ensure clearer expression. Eg;"...because of to the potential...” incorrect; should be "because of the potential..." Please check others.

Response: We thank the reviewer for highlighting this. We have corrected all grammatical issues in the abstract, including “because of to the potential,” now revised to “because of the potential.” [line 14]

- Add briefly the inclusion/exclusion criteria

Response: We added a brief summary of the inclusion/exclusion criteria, noting that the review will include empirical studies published between 2000 and 2024, in English or French, using spatial or spatio-temporal methods for malaria hotspot identification. [line 24]

2. Introduction

- Structure and Logical Coherence

1) Temporal vs. Spatial Heterogeneity: The section starts with a mix of temporal and spatial variation without clearly separating the two concepts. Suggest: Dedicate one paragraph to temporal heterogeneity and another to spatial, then combine to explain spatiotemporal heterogeneity.

2) Flow of Evidence & Citations: While many studies are cited, the relevance of some is not always clearly tied to the narrative. Suggest briefly interpreting what each cited study contributes (e.g., “as shown in Sudan, seasonal variation impacts intervention timing”).

Response: We thank the reviewer for these insightful suggestions. In response, we revised the structure of the introduction by separating the discussion of temporal and spatial heterogeneity into distinct paragraphs. We also ensured that the spatial heterogeneity paragraph provides contextual depth at multiple geographic scales, with each supporting citation explicitly linked to its corresponding spatial level. Additionally, the following paragraph introduces the concept of spatiotemporal heterogeneity to bridge both dimensions and support the rationale for geographically and temporally targeted interventions. [lines 48-63]

- Rationale of the scoping review

1) The rationale is somewhat buried in a long paragraph. This dilutes the impact.

2) You mention that methods have diversified, but not why that matters enough for a dedicated review?

3) Lines 81–85 say the review will “identify and present available information”—this could be more specific. Eg: This review will categorize and compare spatial and spatio-temporal methods, assess common indicators, and highlight trends or inconsistencies in hotspot definitions and applications…..

Response: Thank you for your thoughtful feedback on the clarity and structure of the rationale. We have addressed your suggestions as follows:

1. To improve clarity and impact, we revised the end of the introduction to separate the rationale and the review objectives into two distinct paragraphs. [lines 89-103]
2. We expanded the rationale and now it explicitly explains why the diversity of methods warrants a dedicated scoping review—namely, because this lack of standardization limits comparability, generalizability, and operational relevance for malaria control programs. [lines 84-88]
3. We rewrote the objectives paragraph to be more specific. The review will categorize and compare spatial and spatio-temporal hotspot detection methods, evaluate covariates and indicators used, and highlight trends, inconsistencies, and methodological gaps in the current literature. [lines 99-103]

3. Methods

- *The PCC framework explanation (Population, Concept, Context) is informative, but the subsections would benefit by bullet formatting or clearer subheadings (e.g., bold or italicized "Population", "Concept", "Context") to improve readability.*

Response: We have formatted the PCC elements into distinct bolded subheadings and ensured each subsection is clearly separated for readability.

- *No Boolean operators or filters mentioned*

Response: Although the full Boolean logic structure used in the search has been presented in Appendix 1 of supplementary materials, we went ahead and clearly mentioned the details on the Boolean logic (e.g., AND/OR) used in the methods section. [line 165]

- *Reference to Google Scholar without justification as Google Scholar lacks transparency and indexing control.*

Response: Thank you for pointing this out. We have clarified in the manuscript that Google Scholar was used only during the preliminary keyword exploration phase and was not part of the formal literature search. Only indexed databases with transparent indexing criteria (e.g., PubMed, Scopus) were used for the formal search strategy. [lines 160-163]

- *No mention of publication management software. Add that the results will be imported into Covidence or similar software.*

Response: We have already noted that citations and screening will be managed through Covidence, which is integrated with reference managers. This has now been emphasized in the Study Selection section. [lines 172-173]

- *Exclusion of studies using only GIS visualization- Good decision. Clarify what constitutes "analytical insight" (e.g., statistical modelling vs descriptive mapping)?*

Response: We now explicitly state that "analytical insight" refers to studies using spatial statistical or modeling techniques such as SaTScan, Moran's I, spatial regression, or machine learning—not just descriptive mapping. [lines 147-148]

- *Language limitation to English and French- Reasonable, but mention the rationale; likely due to author capacity or resource constraints.*

Response: We now clarified that the choice was based on the review team's language capacity and preliminary scoping, which showed that most relevant studies are published in English or French. [lines 154-155]

- *"Multidisciplinary studies with only a minor or superficial focus…" - Consider specifying what defines a "superficial focus"; is it word count? lack of quantitative data?*

Response: Thank you for this important suggestion. We have revised the sentence to clarify that studies will be excluded if malaria hotspot identification is not a primary objective or if the term hotspot is only briefly mentioned without any analytical detail. [lines 150-152]

4. Data extraction

- Lack of information on which variables will be extracted Readers and reviewers need to know what type of information the authors plan to synthesize (e.g., study design, modelling techniques, indicators, outcomes).

Response: We thank the reviewer for emphasizing this. In the revised manuscript, we have expanded the Data Extraction section to explicitly describe the full range of variables that will be extracted. In particular, we detailed the information to be collected under "analytical approach," including the type of spatial or spatiotemporal modeling frameworks used, the hotspot detection methods applied (e.g., cluster detection, spatial autocorrelation statistics), data sources, definitions and thresholds for hotspot delineation, and types of outcome and predictor variables analyzed. [lines 188-193]

5. Data analysis and presentation

- The analysis plan feels somewhat scattered and blends narrative, statistical, and visual approaches in one paragraph. Suggestion: Break into clearly labelled subsections for.. Descriptive Analysis, Spatial Visualization, Thematic Synthesis and Implication Mapping

- Consider adding a PRISMA Flow Diagram (if not already planned)

- Please ensure that the data analysis and presentation address the objectives of the scoping review, which include: 1) Identifying and Describing Analytical Methods, 2) Evaluating the Covariates Employed, and 3) Identifying Research Gaps and Recommendations for Future Research.

Response: We thank the reviewer for these valuable suggestions. We have revised the data analysis and presentation section of the manuscript to improve clarity and coherence. Specifically:

- To address the suggestion regarding paragraph structure, we reorganized the analysis plan under clearly labelled subheadings: *Descriptive Analysis*, *Spatial Visualization*, and *Thematic Synthesis*. This new format enhances readability and ensures that each analytical dimension is clearly articulated.
- We confirm that a PRISMA-ScR Flow Diagram will be included in the final review to document the study selection process, in accordance with scoping review reporting standards. A sentence in the Ethics and Dissemination section has also been revised to clarify that the PRISMA-ScR checklist will guide the reporting of this scoping review, not its design or conduct. [lines 224-226]

- To ensure alignment with the review's objectives, we revised the content under each subheading of the section. The whole section now reflects how each objective will be addressed through descriptive summaries, visualizations, and thematic synthesis.

VERSION 2 - REVIEW

Reviewer 1

Name McGee, Kathleen

Affiliation London School of Hygiene & Tropical Medicine

Date 15-May-2025

COI

Thank you for your revisions. This new version of the manuscript is much clearer and robust. Good luck on your next steps. Minor issue that the journal will likely flag: the citations (footnotes) always need to come after the punctuation. Not before. Other than that, I recommend the manuscript be accepted for publication.

Reviewer 2

Name Mahmud , Mohd Amierul Fikri

Affiliation Institute for Public Health, Malaysia, Center for Communicable Disease Research

Date 22-May-2025

COI

All previous comments and concerns have been addressed sufficiently by the authors. The manuscript has been improved accordingly and is now clear, coherent, and scientifically sound. I recommend the manuscript be accepted for publication.
